# A panel of fluorophore-tagged *daf-16* alleles

**DOI:** 10.17912/micropub.biology.000210

**Published:** 2020-01-07

**Authors:** Ulkar Aghayeva, Abhishek Bhattacharya, Oliver Hobert

**Affiliations:** 1 Department of Biological Sciences, Howard Hughes Medical Institute, Columbia University, New York

**Figure 1:**
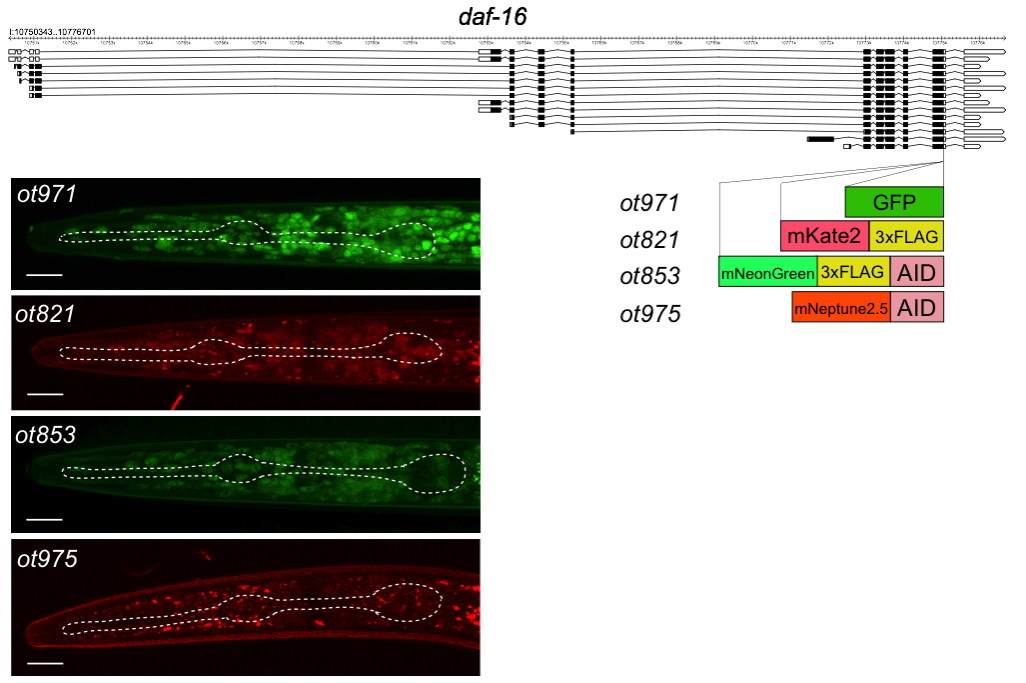
*daf-16* alleles generated by CRISPR/Cas9 genome engineering and their expression patterns in starvation/crowding-induced dauer stage animals*.* The relative signal strength of the image is not directly comparable because different microscope settings had to be used. The absolute signal intensity is GFP>mNeonGreen>mKate2>mNeptune2.5. Scale bars are 10 um.

## Description

*daf-16* encodes a broadly expressed transcription factor that functions in a wide variety of developmental and physiological processes (Lin *et al*., 1997; Ogg *et al.*, 1997; Tissenbaum, 2018), including in the nervous system (Kim and Webb, 2017). DAF-16 protein shows highly dynamic cytoplasmic to nuclear translocation, which, in the past, has been visualized using multicopy constructs, which can produce potential overexpression artifacts (such as those described in (Henderson and Johnson, 2001)). To avoid such overexpression effects, the generation of fluorescently tagged *daf-16* allele would be useful. Similarly, the generation of conditional alleles of *daf-16* would be useful to resolve a number of open questions in regard to the focus of *daf-16* action. To address both issues, we recently generated an mNeonGreen tagged *daf-16* allele that also contained an auxin-inducible degron (Bhattacharya *et al*., 2019; Zhang *et al*., 2015). This allele, *daf-16(ot853[daf-16::mNG::AID]),* enabled us to provide a proof of concept for neuron-type specific *daf-16* depletion (Bhattacharya *et al*., 2019). One issue with this allele has been that due to the emission spectrum of its fluorescent tag (mNeonGreen), it cannot be used in conjunction with *gfp-*based phenotypic read-outs.

We have now genome-engineered three additional fluorophore tagged *daf-16* alleles, some with or without additional tags (FLAG tag or AID degron) (Fig.1). Like the previously published *ot853* allele, we CRISPR/Cas9-engineered the *ot821* allele (*mKate2* tag) with the SEC method (Dickinson *et al*., 2015) and the *ot971* (*gfp* tag) and *ot975* (*mNeptune2.5::AID*) alleles with the so-called Mello method (Dokshin *et al*., 2018), using pDD282 (Dickinson *et al*. 2015) and pEY56 (kindly provided by Eviatar Yemini, based on Addgene plasmid #51310 pcDNA3-mNeptune2.5) as templates. All four alleles show similar expression and subcellular localization patterns. In Figure 1 we show the nuclear localized DAF-16 protein in dauers, generated by a standard crowding/starvation protocol (as described in Bhattacharya *et al*., 2019).

We note that this tagging approach also provides a rare opportunity to assess the intensities of fluorophores side-by-side: We find that GFP expression is brighter than that of mNeonGreen, while mKate2 is clearly weaker and mNeptune2.5 even weaker.

All strains are available at the CGC.

## Reagents

OH16024: *daf-16(ot971[daf-16::gfp])*

OH13908:*daf-16(ot821[daf-16::mKate2::3xFlag])*

OH14125:*daf-16(ot853[daf-16::mNG::AID])*

OH16029: *daf-16(ot975[daf-16::mNeptune2.5::AID])*
